# Efficacy of Revolution^®^ Plus (selamectin plus sarolaner) for the prevention of transmission of *Borrelia burgdorferi* from infected *Ixodes scapularis* to cats

**DOI:** 10.1186/s13071-026-07322-3

**Published:** 2026-03-18

**Authors:** Rachael Isdale, Jamie A. E. Myers, Susan Holzmer, Kaci Shaw, Vickie King, Jessica Y. Rodriguez, Jennifer Hawley, Michael R. Lappin

**Affiliations:** 1 Center for Companion Animal Studies, Department of Clinical Sciences, Colorado State University, Fort Collins, CO USA; 2 Zoetis, Veterinary Medicine Research and Development, Kalamazoo, MI USA

**Keywords:** *Borrelia burgdorferi*, *Anaplasma phagocytophilum*, Acaricide, Tick

## Abstract

**Background:**

*Borrelia burgdorferi* and *Anaplasma phagocytophilum* are transmitted by *Ixodes* spp., with antibodies having been detected in cats in endemic areas. The combination of selamectin plus sarolaner (Revolution^®^ Plus/Stronghold^®^ Plus; Zoetis; RP) is effective against *Ixodes* spp. for 1 month. The objective of this study was to determine whether RP protects cats against transmission of *B. burgdorferi* from *Ixodes scapularis* by killing the ticks before transmission occurs. Transmission of *A. phagocytophilum* was also monitored.

**Methods:**

Ten cats per group were treated once topically either with placebo solution (0.1 ml/kg) or with the minimum label dose of RP (6.0 mg/kg selamectin plus 1.0 mg/kg sarolaner). Thirty days post-treatment, cats were infested with 50 wild-caught adult *I. scapularis*. Ticks were counted, categorized, and removed on day 35. Blood collections for serology occurred on days −6, 30 (prior to infestation), 49, 63, 77, 91, and 104. Serum antibody assay results (*B. burgdorferi* and *A. phagocytophilum*) and polymerase chain reaction (PCR) of skin biopsies (*B. burgdorferi*) were used to define infection rates in the cats.

**Results:**

Treatment with RP resulted in a 100% reduction of *I. scapularis* ticks compared with placebo-treated cats. In placebo-treated cats, antibodies against *B. burgdorferi*, *A. phagocytophilum*, both agents, and *B. burgdorferi* DNA in skin (five, nine, six, and three cats, respectively) were detected by day 104. In contrast, none of the RP-treated cats developed *B. burgdorferi* antibodies or DNA in skin biopsies, and *A. phagocytophilum* antibodies were detected in only two cats, significantly lower than in placebo-treated cats.

**Conclusions:**

Results suggest that a single application of RP at the minimum label dose reduces the risk of infection by both *B. burgdorferi* and *A. phagocytophilum,* when infected at the end of the dosing interval.

**Graphical Abstract:**

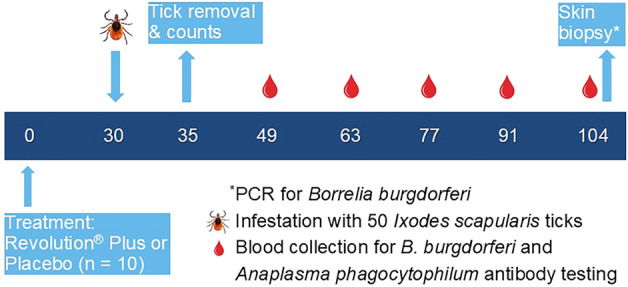

## Background

*Ixodes* species are the primary vectors of *Borrelia burgdorferi* and *Anaplasma phagocytophilum*. Both agents can infect multiple host species, including cats, dogs, and humans [1, 2]. In the Northeast and Midwest regions of the United States, including the regions bordering Canada, these infectious agents are transmitted primarily by *Ixodes scapularis* [1, 3]. In the West region, both agents have been detected in *Ixodes pacificus* [4]. Clinical illness due to *B. burgdorferi* (Lyme disease; borreliosis) or *A. phagocytophilum* (granulocytic anaplasmosis) occasionally occurs and has been well described in people and dogs, however less so in cats [1].

*Ixodes* species are known to infest cats [5–7]. In a nationwide survey of cats with ticks present on physical examination, *I. scapularis* was the most commonly detected species (53.1%). In one survey of adult *I. scapularis* ticks, 25.7% were positive for *B. burgdorferi* DNA by polymerase chain reaction (PCR) [7]. Using an enzyme-linked immunosorbent assay (ELISA) titrated for use with canine sera (SNAP 3Dx; IDEXX Laboratories), antibodies against *B. burgdorferi* were first detected in the serum of naturally exposed, client-owned cats in the USA in 1990 [5]. Additional studies in the Northeast USA and Europe provided further data documenting that cats are exposed to *B. burgdorferi* and that they mount a response to the C6 peptide used in the assay [8–10].

Since *A. phagocytophilum* and *B. burgdorferi* share the same vector and induce positive antibody and PCR test results even in the absence of clinical signs, it has been difficult to determine which of the agents is responsible for disease, if at all, in individual cats. However, field studies of cats in Maine (USA), Germany, and Scotland provide evidence for clinical borreliosis in at least some cats [10–13]. It is also important to note that evidence of exposure or infection with multiple tick-borne pathogens in cats has included *Bartonella* species, *Borrelia miyamotoi*, *Ehrlichia* spp., *Francisella tularensis*, and the hemotropic *Mycoplasma*, which could potentiate or confound the attribution of the primary cause of disease when *B. burgdorferi* and *A. phagocytophilum* are detected [1, 3, 11, 14, 15]. These data all suggest that using tick control on cats is likely to have health benefits to the cat and other family members [16].

The first objective of the present study was to evaluate the efficacy of the combination of selamectin plus sarolaner (Revolution^®^ Plus/Stronghold^®^ Plus, Zoetis Inc., Parsippany, NJ; RP) in preventing the transmission of *B. burgdorferi* at the minimum commercial dose when compared to placebo in cats. The second objective was to gather more data on potential development of clinical signs of feline borreliosis.

## Methods

### General study design and animal management

Nine female and 11 neutered male cats, approximately 3 years old, were identified by unique ear tattoos and individually housed in one room throughout the study to avoid potential transfer of RP between cat groups or the very unlikely event of direct transmission of infectious agents. Prior to the start of the study (day −8), the cats were physically normal as determined by a veterinarian and weighed 3.0–7.1 kg. The cats had been raised since birth indoors in vector-naïve environments and were negative for antibodies against *Anaplasma* species*, B. burgdorferi*, and *Ehrlichia* species as well as *D. immitis* antigen (SNAP^®^ 4DX^®^ Plus; IDEXX) when selected for the study. The cats were fed commercially available adult cat food and were provided water ad libitum throughout the study.

The cats were randomly allocated to treatments and pens according to a completely randomized design by the biometrician. RP-treated and placebo-treated groups included 10 cats per group. The general study design was based on a previous study assessing preventive efficacy of oral sarolaner in dogs against *B. burgdorferi* at the end of the dosing interval [17]*.* The following was the overall schedule of key events that are described in detail in individual sections:

- Day −8 to day 103: clinical monitoring of all study cats
- Day −7 to day 0: acclimatization period
- Day 0: treatment with RP or placebo
- Day 30: infestation with adult *I. scapularis*
- Day 35: tick removal and counts
- Days 49, 63, 77, 91, and 104: blood collection
- Day 104: blood collection and skin biopsies

To maintain masking, study personnel making observations, conducting post-treatment tick infestations or post-treatment tick counts, or conducting general care for the cats were masked to the treatment groups. General health observations were made by appropriately trained facility personnel twice daily up to day 34, with the exception of day 0, when four clinical observations were conducted, and on day 1, when one clinical observation was conducted. RP was applied topically at the base of the skull between the shoulder blades, according to the product label. After cats were treated on day 0 according to the allotment, the cats were observed for systemic and local effects at 1 h (±15 min), 3 h (±30 min), 6 h (±1 h), and 24 h (±1 h) after dosing. From days 35 to 104, the cats had general health assessed once daily, and clinical examinations were conducted at a separate time to assess for clinical signs of tick-borne disease which included but were not limited to ataxia, hyporexia, lameness, and lethargy. If these clinical signs were noted, the study veterinarian was to perform a complete physical examination, including but not limited to rectal body temperature.

On day 0, either RP at the minimum label dose of 1.0 mg/kg sarolaner plus 6.0 mg/kg selamectin (at a volume of 0.1 ml/kg) or 0.1 ml/kg placebo was administered topically to the appropriate cats. The placebo treatment was a topical solution containing the same excipients as RP without the active ingredients. From day 0 until the end of the study, separate outer garments, disposable gloves, and equipment were used for each cat to prevent cross-contamination.

#### Tick infestation and removal

The adult *I. scapularis* were wild-caught in field locations in South Kingstown (RI, USA) known to be endemic for *B. burgdorferi* approximately 2 months before the start of the study. A random representative sample of the ticks was assessed by PCR, which showed approximate infection rates of 60% for *B. burgdorferi*, 15% for *A. phagocytophilum*, and 10% for *Babesia microti*. The ticks that were supplied for the infestations were pre-counted into individual vials containing 50 (±5) unfed adult *I. scapularis* using a sex ratio as close as possible to 1:1 as determined by the specialist providing the ticks. A study investigator (MRL) confirmed the viability of the ticks immediately prior to infestation.

On day 30, each cat was sedated per the facility protocol and then infested with *I. scapularis* by applying one vial of 50 ticks to each cat directly by tapping it onto the hair coat of the cat. A soft Elizabethan collar was applied to the neck of each cat after the infestation to prevent grooming. On days 30–35, the collars were checked at least twice daily.

On day 35, all *I. scapularis* were manually removed using forceps and/or flea combs and categorized as either live or killed, free-roaming, attached unengorged, or attached engorged. Instruments were cleaned between animals to prevent cross-contamination. Areas with attached *I. scapularis* were recorded. The four areas of the body with the highest number of attached ticks or the area(s) with the most notable inflammation from attached ticks, as determined by visual inspection, or the four most common sites of tick attachment if neither of the former were identified, were shaved on each cat. If an area of the face, ears, or head was one of the identified sites, an area of the cat’s neck close to the site(s) of tick attachment was shaved to reduce discomfort to the animal. If fewer than four sites of tick attachment were present on a cat, the four most common areas of tick attachment from cats with infestations were shaved. The sites were shaved periodically to keep sites identifiable up to day 104 in preparation for skin biopsies.

#### Sample collection and assays

On days 49, 63, 77, 91, and 104, blood samples (2 ml in ethylenediaminetetraacetic acid [EDTA] for PCR assays if indicated; 3 ml in a clot tube for serum separation for antibody testing) were collected and assayed as described, and the remnant samples stored at −80 °C. On day 104, the cats were sedated, and two 4-mm skin biopsies were obtained from the shaved regions where *I. scapularis* had been attached.

All sera were assayed with a commercially available kit titrated for use with canine sera that qualitatively provides results for *B. burgdorferi*, *A. phagocytophilum*/*Anaplasma platys*, and *Ehrlichia canis*/*Ehrlichia ewingii* antibodies and *D. immitis* antigen (SNAP^®^ 4Dx^®^ Plus, IDEXX, Westbrook, ME, USA). DNA was extracted for one set of skin biopsy samples for each cat and assayed for *B. burgdorferi* DNA in a commercial laboratory (New York State Veterinary Diagnostic Laboratory) and in the laboratory of one of the study investigators (MRL), both using previously published techniques [18, 19]. Cats were considered positive for *B. burgdorferi* by skin biopsy if at least one of the two PCR methods detected DNA.

#### Statistical methods

An adequate *I. scapularis* rate was defined as having at least 6 of 10 placebo-treated cats with 12 or more live attached ticks on day 35. Live tick counts were analyzed with a general linear mixed model with the fixed effect of treatment and random effect of residual. The presence of *B. burgdorferi* or *Anaplasma* species antibodies in serum was considered evidence of infection by the agents. Amplification of *B. burgdorferi* DNA by either PCR assay was also considered evidence of infection. Infection rates between the two groups were compared by Fisher’s exact test. The presence of *Anaplasma* species antibodies in serum was analyzed with a generalized linear mixed model using a binomial distribution and logit link function. The fixed effect was treatment. All hypothesis tests were conducted at the 0.05 level of significance (two-tailed).

## Results

### Clinical observations

There was no evidence of local irritation or systemic reactions due to treatment noted in the first 24 h after administration of the placebo or RP. Vomiting or diarrhea was the most common adverse event reported in both treatment groups (six cats and one cat, respectively, in the placebo group; seven cats and one cat, respectively, in the RP group) and was intermittent and mild. None of the cats developed clinical signs of tick-borne disease during the course of the study.

#### Tick attachment rates and efficacy of Revolution Plus against ticks

Each cat in the placebo-treated group had attached, live *I. scapularis* on day 35 (Table 1), with numbers ranging from 8 to 24 (mean = 15.2 per cat). Since at least six of the cats had 12 or more attached *I. scapularis*, the challenge was considered successful. Since none of the RP-treated cats had attached live ticks, the reduction was 100% (*P* < 0.0001).

**Table 1:** Summary of live tick counts for individual cats with means, ranges, and percent reduction on study day 35 (5 days post-infestation)

Treatment	Cat no.	Tick count
Placebo	1	13
	2	11
	3	20
	4	17
	5	8
	6	18
	7	9
	8	24
	9	15
	10	17
	Range	8–24
	Least-squares mean	15.2
Revolution Plus (sarolaner + selamectin)	1	0
	2	0
	3	0
	4	0
	5	0
	6	0
	7	0
	8	0
	9	0
	10	0
	Least-squares mean	0.0
	% reduction	100
	*P* value	< 0.0001

#### *Anaplasma phagocytophilum* and *Borrelia burgdorferi* results

None of the cats in the RP treatment group developed detectable *B. burgdorferi* antibodies over the course of the study nor had *B. burgdorferi* DNA amplified from the skin biopsies collected on day 104 by either PCR assay. In contrast, *B. burgdorferi* was detected in a total of six placebo-treated cats. Antibodies were detected in sera from five placebo-treated cats, of which two cats were also positive for DNA in skin. Furthermore, DNA was found in the skin biopsy of one placebo-treated cat that never developed detectable antibodies (Table 2). The difference between groups for *B. burgdorferi* infection was significant (*P* = 0.0108), and the efficacy of the product for blocking infection was 100%. *Borrelia burgdorferi* antibodies were detected as early as day 49 (19 days after tick placement) or as late as day 91 (61 days after tick placement).

**Table 2:** *Anaplasma phagocytophilum* and *Borrelia burgdorferi* results in placebo-treated cats infested with wild-caught *Ixodes scapularis* (25 male/25 female) per cat on day 30

Cat no.	*Ap* AB days	*Bb* AB days	*Bb* PCR skin	Infection^a^
1	63, 77, 91, 104	Negative	Negative	*Ap*
2	Negative	Negative	Negative	None
3	49, 63, 77, 91, 104	77, 91, 104	Positive	*Ap*/*Bb*
4	63, 77, 91, 104	Negative	Positive	*Ap*/*Bb*
5	49, 63, 77, 91, 104	49, 63, 77, 91, 104	Negative	*Ap*/*Bb*
6	49, 63, 77, 91, 104	63, 77, 91, 104	Positive	*Ap*/*Bb*
7	63, 77, 91, 104	Negative	Negative	*Ap*
8	77, 91, 104	91, 104	Negative	*Ap*/*Bb*
9	63, 77, 91, 104	Negative	Negative	*Ap*
10	63, 77, 91, 104	63, 77, 91, 104	Negative	*Ap*/*Bb*

_a_ At least one positive serology or *Bb* DNA in the skin on day 104 (results of both PCR assays combined)

*I. scapularis* placed on day 30 and removed on day 35

Abbreviations: AB = antibody; *Ap* = *A. phagocytophilum*; *Bb* = *B. burgdorferi*

*Anaplasma phagocytophilum* antibodies were detected in sera on at least one time point from nine cats in the placebo-treated group (Table 2) and two cats (Table 3) in the active treatment group, which was significantly different (*P* = 0.0141). The two cats in the RP group were positive either once on day 63 or on days 63, 77, 91, and 104. Overall, *A. phagocytophilum* antibodies were first detected as early as day 49 (19 days after tick placement) or as late as day 77 (37 days after tick placement).

**Table 3:** *Anaplasma phagocytophilum* and *Borrelia burgdorferi* results in Revolution Plus-treated cats infested with wild-caught *Ixodes scapularis* (25 male/25 female) per cat on day 30

Cat no.	*Ap* AB days	*Bb* AB days	*Bb* PCR skin	Infection^a^
1	Negative	Negative	Negative	None
2	Negative	Negative	Negative	None
3	Negative	Negative	Negative	None
4	63^b^	Negative	Negative	*Ap*
5	Negative	Negative	Negative	None
6	Negative	Negative	Negative	None
7	63, 77, 91, 104	Negative	Negative	*Ap*
8	Negative	Negative	Negative	None
9	Negative	Negative	Negative	None
10	Negative	Negative	Negative	None

_a_ At least one positive serology or *Bb* DNA in the skin on day 104 (results of both PCR assays combined)

_b_Reserved serum sample was negative on retest

*Ixodes scapularis* placed on day 30 and removed on day 35

Abbreviations: AB = antibody; *Ap* = *A. phagocytophilum*; *Bb* = *B. burgdorferi*

## Discussion

The results of this study demonstrate that a single application of RP at the minimum label dose resulted in a 100% reduction of *I. scapularis* ticks using the model described here. This reduction is consistent with what has been previously demonstrated with this product in cats against *I. scapularis* and other *Ixodes* species when evaluated at 48 h after tick placement at the end of the dosing interval [20, 21].

Infection proportions of the ticks in this study, with 60% carrying *B. burgdorferi* and 15% positive for *A. phagocytophilum*, were similar to another study [6]. Since none of the RP-treated cats developed evidence of *B. burgdorferi* infection, the single application of the product presumably killed the *I. scapularis* before *B. burgdorferi* could be transmitted when ticks were applied on day 30. It should be noted that according to the World Association for the Advancement of Veterinary Parasitology (WAAVP) guidelines for studies evaluating the efficacy of parasiticides in reducing the risk of vector-borne transmission in dogs and cats, the recommendation in laboratory studies is to challenge animals at least at the beginning and the end of the claim duration [22]. In this study, cats were challenged only at the end of the dosing interval using a study design previously used in dogs with oral sarolaner [17]. For RP, plasma concentrations of sarolaner at 24 h and 28 days post-dose are comparable (internal data).

As noted in the results, more cats in the placebo-treated group of this study were ultimately infected by *A. phagocytophilum* (nine cats) than by *B. burgdorferi* (six cats), even though the tick infection proportions were lower. These results emphasize how transmissible *A. phagocytophilum* infection is to cats and may explain why there are more studies showing positive results for this agent in cats than for *B. burgdorferi* [12, 23–26]. Only two cats in the RP treatment group developed evidence of *A. phagocytophilum* transmission. One RP treatment cat was positive for *A. phagocytophilum* only on day 63, which suggests either a short-lived, immune-cleared infection or a false positive result. When stored serum was retested, the sample was negative for *A. phagocytophilum* antibodies and also negative for DNA in stored whole blood by PCR assay [19]. However, even when this cat was included in the final analysis, the single treatment with RP showed significant protection (*P* = 0.0141) against *A. phagocytophilum* compared to the placebo-treated group. The time required for tick attachment to allow for transmission by adult *I. scapularis* is likely between 12 and 24 h [27–29]. These results suggest that RP acaricidal activity affected the adult *I. scapularis* quickly enough to either block *A. phagocytophilum* transmission completely or limit the transmission of the pathogen in this experimental model. It should be noted that the use of 50 ticks in this model can be considered as a worst-case scenario, as the tick infection rate exceeds what is described on most cats in the USA [7]. The 15% infection rate with *A. phagocytophilum* resulted in a 90% infection rate in placebo-treated animals, again underpinning a severe scenario testing in this infection model.

The lack of clinical signs consistent with systemic illness with either *B. burgdorferi* or *A. phagocytophilum* in this study was not surprising. None of the small number of experimentally infected cats in a previous study developed clinically manifested illness [6]. In contrast, when clinical illness is recognized in client-owned, naturally exposed cats with suspected borreliosis or granulocytic anaplasmosis, fever, lethargy, and inappetence are commonly reported [12, 23–25]. The differences between laboratory and field studies may merely reflect the small number of exposed cats in the laboratory studies, longer duration of infections in natural field infections, or the more diverse immune and general health status of cats in field studies.

Another limitation to the study described here was the use of an assay titrated for use with dog sera as one of the criteria to define the infection status of individual cats. While this assay has been used to detect both *B. burgdorferi* and *A. phagocytophilum* antibodies in cat sera after infestations with *I. scapularis* ticks, the use of an optimized assay should be considered in the future [6]. Using this canine assay, seroconversion after *I. scapularis* attachment was delayed for both *A. phagocytophilum* (37 days) and *B. burgdorferi* (61 days), which limits the clinical use of this assay in assessing cats with acute illness.

While *A. phagocytophilum* DNA is frequently amplified by PCR in blood during the acute clinical and experimental infections, the DNA of *B. burgdorferi* is quickly cleared from blood [6, 23–25, 30]. However, *B. burgdorferi* DNA can persist in the sites of tick attachment and organism transfer, which is used as another criterion to document transmission by this agent.

Only two of the five placebo cats that were antibody-positive were also PCR-positive for *B. burgdorferi* on the skin biopsies. In contrast, dog experimental infection studies tend to show a high correlation between antibody-positive and PCR-positive samples. For example, in one previous study assessing the ability of sarolaner (Simparica^®^, Zoetis Inc., Parsippany, NJ, USA) to prevent transmission of *B. burgdorferi* in dogs, five out of six dogs in the placebo group that were antibody-positive were also positive by skin PCR [17]. These data suggest that cats may have a better immune mechanism than dogs for clearing *B. burgdorferi* from tissues.

## Conclusions

A single topical application of RP at the minimum label dose of 6.0 mg selamectin and 1.0 mg sarolaner per kilogram body weight provided 100% efficacy in preventing transmission of *B. burgdorferi* and significantly reduced transmission of *A. phagocytophilum* in cats when wild-caught *I. scapularis* ticks were infested on day 30 post-treatment.

## Data Availability

The dataset supporting the conclusions of this article is included within the article.

## Abbreviations

EDTA: Ethylenediaminetetraacetic acid
ELISA: Enzyme-linked immunosorbent assay
PCR: Polymerase chain reaction
RP: Revolution Plus
